# Progress in Medicine

**Published:** 1897-11-13

**Authors:** 


### Progress in Medicine.

### OBSTETRICS.

(*Continued from page* 100.)

**Cæsarian Section.**—B. C. Hirst[22] describes the technique which he uses in cases of Cæsarian section. He considers the exciting of labour pains before operation unnecessary. He administers strychnine, digitalis, and quinine, in pill three times a day before operation, and secures purgation by salines; gives sulphonal, 10 grains, on the evening of the day preceding in nervous patients, and also half an ounce of Epsom salts. After careful cleansing of the patient's abdomen and the operator's hands and arms, the abdominal wall is incised from just above the symphysis to two inches above the umbilicus, the incision enlarged if necessary, and the uterus delivered. The abdominal wound being pressed closely around the cervix, an incision one inch in length is made through the uterine wall, and the uterus torn open with the fingers down to the internal os, the membranes ruptured, and the fœtus delivered. This is followed by hysterectomy, with the formation of peritoneal flaps, ligature of the uterus, sewing of the flaps over the stump, and the dropping of the stump into the pelvis, followed by closure of the abdominal wound.

Successful cases of Cæsarian section are reported by Ward Cousins[23] and Bèchet.[24] Cousins' case was performed after unsuccessful craniotomy in a patient with a conjugate diameter of 2½ in. and transverse of 3 in. The uterus was not delivered, and the uterine wound closed by catgut sutures. The abdominal wound was closed by three rows of catgut and a superficial row of silk sutures. Bèchet's case was performed at the patient's home. The uterine incision passed through the placental site, but the resulting hæmorrhage was checked by injection of ergotin and application of compresses of hot sterilised water. The uterine muscle and peritoneum were sutured separately, and the abdomen closed. Robert Sorel[25] reports a case of Cæsarian section in a case where labour, which had lasted 60 hours, was obstructed by inoperable cancer, no dilatation having occurred at the end of that time. The child was saved alive, and the mother recovered. The cancer, however, which had invaded the vagina, steadily progressed. The vaginal invasion prevented complete hysterectomy.

**Pregnancy with Ovarian Tumour.**—In dealing with ovarian tumour complicating pregnancy, Sir John Williams[26] alludes to the high mortality following this complication, and deals, firstly, with the question, "Is pregnancy a cause of ovarian tumours?" He shows that, age for age, ovarian tumour is far less frequent in the married than in the single, basing this result on the statistics of 1,000 operations by Spencer Wells; also that the ratio decreases in favour of the married as age advances. He considers that, for this purpose, any error caused by regarding all married women as mothers is balanced by the error possible in regarding all the unmarried as nulliparous. He notes from his own collection of cases that one-third of the total number occurred in primiparæ, while the proportion diminished as the number of pregnancies advanced. He also shows that as a rule, apart from accidents such as twisting of the pedicle, the rate of growth is unaffected by pregnancy or parturition.

He then shows that the influence of ovarian tumour on parturition is to cause an enormous puerperal mortality, but not a large mortality during pregnancy if not interfered with. He concludes that to prevent this two courses are open: ovariotomy during pregnancy, the success of which is proved, and the anticipating of labour by Cæsarian section in cases where adhesions prevent the removal of the tumour. The mortality after ovariotomy during pregnancy is less than one-half that of normal labour complicated by the presence of ovarian tumour.

A case of ovarian tumour complicating labour is reported by Riddell[27]; the tumour was pushed up into the abdomen and the child delivered with forceps. The tumour, removed later by abdominal section, proved to be a divisional cyst. The patient was three and a half months pregnant at the time of operation for removal of the cyst, and recovered.

J. D. Jones[28] reports a case of strangulated ovarian cyst complicating pregnancy; the patient was admitted with symptoms of peritonitis, and a globular swelling as large as the adult head was found in the left hypochondrium. The abdomen was opened over the tumour, and an ovarian cyst, black from strangulation of its pedicle by twisting, discovered. It was removed, and the pedicle ligatured through very questionable tissues close to the left uterine cornu. The stump was surrounded by gauze, and brought out of the lower angle of the abdominal wound, but suppurated after five or six days. Twenty-four days after the operation the patient had a rigor and high temperature, and miscarried. The suppuration ceased a few days later, and the patient recovered completely.

**Pregnancy with Fibroids.**—In treating of fibroids complicating pregnancy, Vanderveer[29] considers that suprapubic hysterectomy is the safest course if at or near term dyotocia threatens; while the same treatment should be adopted earlier if the tumour was growing rapidly, since myomectomy being inadmissible, and the high mortality after abortion in these cases, rendered it necessary to act promptly.

**Albuminuria and Eclampsia in Pregnancy.**—Clifford Allbutt[30] states, with reference to albuminuria in pregnancy, that the theories which relate to the mechanical causation of this condition, *e.g.*, by pressure of the growing uterus, cannot be sustained, and contrasts the state of the kidneys post-mortem in a case of cardiac disease, where alterations of their structure are not such that relief of the condition causing them fails to re-establish their suspended functions, with the condition in a fatal case of eclampsia where acute inflammatory processes are found; and also that complete thrombosis of the renal veins does not produce eclampsia, which does not occur, moreover, in the case of large fibroid or ovarian tumours.

He adduces strong arguments in favour of the causation of this condition by a toxin normally excreted by the urine. Urine is found to be toxic when healthy and filtered, if injected into the circulation, while the urine of a puerperal woman suffering from albuminuria is not more but less so; the serum of such a patient, on the other hand, is twice as poisonous, weight for weight. The serum of the fœtus in such a case is also poisonous in about the same ratio (2—1) as compared with healthy serum. He discusses the bacillary with other theories, and dismisses it as unproven. He shows that the fœtus is a great source of toxins. Hence he emphasises the avoidance of gastro-intestinal catarrh by careful dieting and the necessity for terminating pregnancy. He considers that a certain amount of immunity is conferred after a first attack, although the albuminaria may recur, and cites the large proportion of primiparæ to multiparæ attacked as some evidence of this.

Christian Simpson[31] adduces further evidence of toxin poisoning in the occurrence of salivation in pregnancy, and shows that this is also due to a toxin excreted by the urine, and present in large quantity in blood, liver, and muscle.

The sources of auto-intoxication are shown to depend on a combination of hepatic derangement and intestinal decomposition with renal inadequacy. Either condition may be primary. The toxicity of bile and serum is diminished by decoloration; hence, charcoal and intestinal antiseptics are of value, and the treatment by hydragogue purgatives is shown to rest on a sound basis. The blood is the source of the toxin, and contains it largely. Thirty-two grammes of blood contain 50 centigrammes of extractives, so that the loss of one ounce of blood will eliminate 7½ grains of extractives, or one-sixteenth of the daily total excreted by the urine. Hence, bleeding is an efficient remedy, as it rapidly removes extractives.

Clark and Kelton[32] consider that more attention than they deserve has been given to the kidneys, and that the theory of the renal production is untenable; that attention to the renal secretions alone is inadequate; that the poison depends on insufficient hepatic action; and that attention to the liver will compensate for renal insufficiency. That the nephritis is secondary, and analogous to that of scarlatina.

Grandin[33] recommends in cases where the action of the kidneys is suspended after delivery of the child, or abdominal operation, the irrigation of the intestine with a 1 per cent. solution of salt. The buttocks are elevated and a large rectal tube passed as far as possible; an attendant should hold the rectal tube close to the anus to prevent its expulsion as the water is driven out. The temperature of the water should be 188 deg. Fahr. (*sic*) [this must be a mistake for 108 deg. or 118 deg.], and eight to ten gallons allowed to flow in from a gravitation can. Croton oil should meanwhile be administered, and the patient wrapped in blankets. Profuse diaphoresis sets in, catharsis ensues, and the kidneys recommence to secrete.

Solé[34] recommends large subcutaneous injections of saline solution, and reports a case in which he injected a pint of the solution on one occasion, and later half a pint into the subcutaneous tissue of the axillæ and flank with success. He states that this should not be done if acute inflammation of the kidney is present.

W. H. Thayer[35] claims that in eclampsia a condition of the nervous system is present which causes a peculiar tolerance of certain drugs, especially of veratrum viride. The tincture may be administered in a dose of a drachm, and if ineffectual at first, may be repeated in smaller doses. The pulse should be carefully watched, and while it is kept below 60 the convulsions do not return. Collapse and vomiting, if caused by the drug, may be combated by the administration of opium or of morphia hypodermically, or by the administration of a diffusible stimulant. The effects produced are cooling of the surface and lowering of the pulse in rate but not in strength, together with arrest of the convulsions.

Macalister[36] reports a case of eclampsia treated by inhalation of oxygen; the patient was a primipara, aged 31. She was seven months pregnant, and had three eclamptic seizures before treatment was commenced, in one of which two ounces of urine was drawn off and found to be nearly solid with albumen. Lividity and dyspnœa being extreme, the inhalation of oxygen was commenced on the appearance of the fourth fit, and was followed by a marked diminution in the violence of the convulsive movements and cessation of lividity. The administration was continued in the fits which followed with equally good effect. She was delivered of a dead child on the day following the first fit. The convulsions persisted, but the attacks were shorter and the coma gradually became less. She became conscious on the third day, but had three convulsive attacks with two on each of the two succeeding days, after which she made a good recovery. The inhalations were most successful in combating the lividity and dyspnœa which led to their use. Two injections of Pilocarpin were followed by a convulsion and vomiting, while the passage of the catheter and the administration of a vaginal douche were also followed by convulsions. This method of treatment seems well worthy of further trial. It can be used as a supplementary measure to other remedies, with which t would not interfere in any way.

[22] American Journ. of Obstetrics, Jan., 1897, quoted in Therapeutic Gazette, April 15, 1897. [23] B. M. J., May 1, 1897. [24] Amer. Journ. Med. Science, March 1897, quoted from Le Progrès Medicale, 1896, No. 46. [25] Arch. Provinc de Chirurg., quoted in Epit. B. M. J., June 12, 1897. [26] Lancet, July 17, 1897. [27] Scottish Med. and Surg. Journ., No. 2, 1897, quoted in Amer. Journ. Med. Science, May, 1897. [28] New York Med. Record, April 20, 1897. [29] New York Med. Record, Nov. 20, 1896. [30] Lancet, Feb. 27, 1897. [31] Lancet, July 10, 1897. [32] Quoted in Amer. Med.-Surg. Bulletin, Aug. 10, 1897. [33] Therapeutic Gazette, F b. 15, 1897. [34] Therapeutic Gazette, June, 1897, quoting from Le Presse Medicale Belgique. [35] Clinical Journal, May 13, 1897. [36] Lancet, July 17, 1897.

